# Supplementary material for: Genetic Predictors of Psychosomatic Symptoms in Individuals with Metabolic Syndrome: Insights from a Cross-Sectional Study in Kazakhstan
Source: Int J Environ Res Public Health. 2025 Dec 12;22(12):1853. doi: 10.3390/ijerph22121853 (PMC12733059; doi:10.3390/ijerph22121853)
Supplement: Supplementary file 1 [file ijerph-22-01853-s001.zip › ijerph-3977034-supplementary.pdf]

**Supplementary Table 1.** Results of the test for normality of distribution among variables of interest and chosen test type for further analysis.

| | Metabolic syndrome (1=yes; 0=no) | Gene | Genotype | Psychosomatic indicator | Number of observations | Skewness | Kurtosis | P-value for Shapiro-Wilk test | P-value for Kolmogorov-Smirnov test | Chosen test type |
|---|---|---|---|---|---|---|---|---|---|---|
| 1 | 1 | LPL* | AA | Anxiety | 41 | -0.904 | 3.013 | 0.001693 | 0.02672 | Kruskal-Wallis |
| | 1 | LPL | GA | Anxiety | 29 | 0.144 | 3.294 | 0.284797 | 0.65595 | |
| | 1 | LPL | GG | Anxiety | 11 | -0.271 | 1.705 | 0.113937 | 0.77575 | |
| 2 | 1 | LPL | AA | Depression | 41 | 0.013 | 2.21 | 0.591415 | 0.79086 | ANOVA |
| | 1 | LPL | GA | Depression | 29 | 0.471 | 3.158 | 0.232507 | 0.55028 | |
| | 1 | LPL | GG | Depression | 11 | -0.392 | 2.521 | 0.923604 | 0.98007 | |
| 3 | 1 | LPL | AA | Stress | 41 | 0.137 | 2.344 | 0.180941 | 0.50465 | ANOVA |
| | 1 | LPL | GA | Stress | 29 | -0.411 | 2.565 | 0.145668 | 0.24868 | |
| | 1 | LPL | GG | Stress | 11 | -0.153 | 1.778 | 0.67488 | 0.99513 | |
| 4 | 1 | NPY # | CC | Anxiety | 17 | 0.195 | 4.082 | 0.157611 | 0.77674 | Kruskal-Wallis |
| | 1 | NPY | TC | Anxiety | 49 | -0.316 | 2.761 | 0.013959 | 0.07555 | |
| | 1 | NPY | TT | Anxiety | 17 | -0.533 | 2.341 | 0.220588 | 0.63884 | |
| 5 | 1 | NPY | CC | Depression | 17 | -0.071 | 2.606 | 0.475163 | 0.49864 | ANOVA |
| | 1 | NPY | TC | Depression | 49 | 0.133 | 2.63 | 0.442517 | 0.64462 | |
| | 1 | NPY | TT | Depression | 17 | -0.211 | 2.055 | 0.709352 | 0.83465 | |
| 6 | 1 | NPY | CC | Stress | 17 | 0.169 | 2.059 | 0.434912 | 0.90961 | ANOVA |
| | 1 | NPY | TC | Stress | 49 | -0.224 | 2.193 | 0.206468 | 0.59654 | |
| | 1 | NPY | TT | Stress | 17 | 0.568 | 3.177 | 0.174162 | 0.47787 | |
| 7 | 0 | LPL | AA | Anxiety | 54 | -0.194 | 1.778 | 0.004646 | 0.2847 | Kruskal-Wallis |
| | 0 | LPL | GA | Anxiety | 48 | -0.347 | 3.042 | 0.13548 | 0.34324 | |
| | 0 | LPL | GG | Anxiety | 8 | 1.47 | 4.117 | 0.043219 | 0.75577 | |
| 8 | 0 | LPL | AA | Depression | 54 | 0.236 | 2.012 | 0.020906 | 0.19836 | Kruskal-Wallis |
| | 0 | LPL | GA | Depression | 48 | -0.108 | 2.337 | 0.322078 | 0.7559 | |
| | 0 | LPL | GG | Depression | 8 | 0.495 | 1.965 | 0.486174 | 0.77583 | |
| 9 | 0 | LPL | AA | Stress | 54 | 0.12 | 2.402 | 0.541011 | 0.73414 | ANOVA |
| | 0 | LPL | GA | Stress | 48 | -0.396 | 2.754 | 0.066021 | 0.2204 | |
| | 0 | LPL | GG | Stress | 8 | 0.548 | 2.695 | 0.833659 | 0.96418 | |
| 10 | 0 | NPY | CC | Anxiety | 19 | -0.107 | 1.587 | 0.055884 | 0.48093 | Kruskal-Wallis |
| | 0 | NPY | TC | Anxiety | 66 | -0.199 | 2.101 | 0.021963 | 0.34278 | |
| | 0 | NPY | TT | Anxiety | 24 | -0.301 | 2.875 | 0.246107 | 0.3394 | |
| 11 | 0 | NPY | CC | Depression | 19 | 0.34 | 1.772 | 0.057163 | 0.58099 | ANOVA |
| | 0 | NPY | TC | Depression | 66 | -0.033 | 2.291 | 0.247043 | 0.39619 | |

| | | | | | | | | | | |
|---|---|---|---|---|---|---|---|---|---|---|
| | 0 | NPY | TT | Depression | 24 | -0.29 | 2.34 | 0.248802 | 0.62535 | |
| **12** | 0 | NPY | CC | Stress | 19 | -0.064 | 1.825 | 0.2866 | 0.85274 | ANOVA |
| | 0 | NPY | TC | Stress | 66 | -0.202 | 2.518 | 0.218952 | 0.55833 | |
| | 0 | NPY | TT | Stress | 24 | -0.134 | 2.734 | 0.628413 | 0.92686 | |
